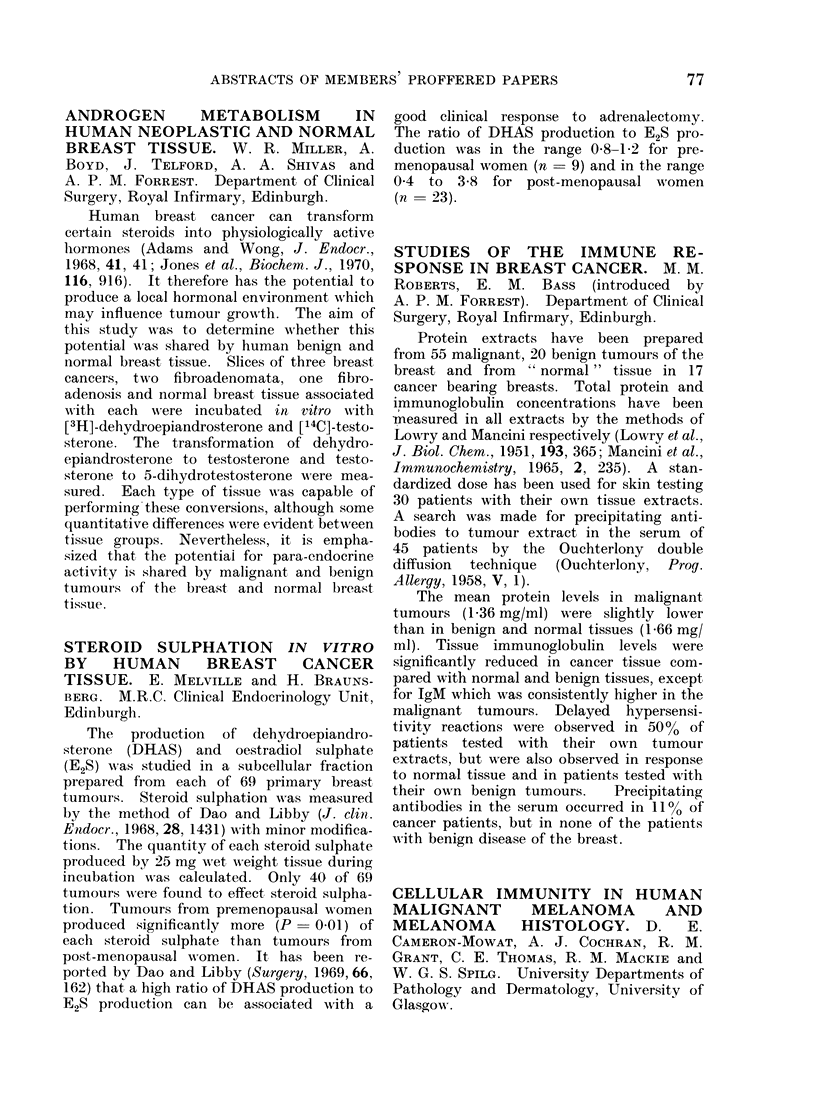# Steroid sulphation in vitro by human breast cancer tissue.

**DOI:** 10.1038/bjc.1973.77

**Published:** 1973-07

**Authors:** E. Melville, H. Braunsberg


**STEROID SULPHATION *IN VITRO* BY HUMAN BREAST CANCER TISSUE.** E. MELVILLE and H. BRAUNSBERG. M.R.C. Clinical Endocrinology Unit, Edinburgh.

The production of dehydroepiandrosterone (DHAS) and oestradiol sulphate ($E_2S$) was studied in a subcellular fraction prepared from each of 69 primary breast tumours. Steroid sulphation was measured by the method of Dao and Libby (*J. clin. Endocr.*, 1968, **28**, 1431) with minor modifications. The quantity of each steroid sulphate produced by 25 mg wet weight tissue during incubation was calculated. Only 40 of 69 tumours were found to effect steroid sulphation. Tumours from premenopausal women produced significantly more ($P = 0{\cdot}01$) of each steroid sulphate than tumours from post-menopausal women. It has been reported by Dao and Libby (*Surgery*, 1969, **66**, 162) that a high ratio of DHAS production to $E_2S$ production can be associated with a good clinical response to adrenalectomy. The ratio of DHAS production to $E_2S$ production was in the range 0·8–1·2 for premenopausal women ($n = 9$) and in the range 0·4 to 3·8 for post-menopausal women ($n = 23$).